# Case Report: Rapid loss of endogenous insulin secretion and reversible subcutaneous insulin resistance following exogenous insulin exposure in a patient initially diagnosed with type 2 diabetes

**DOI:** 10.3389/fendo.2026.1962271

**Published:** 2026-09-17

**Authors:** Si Wen, Zongjun Chen, Yanxia Hu, Feiya Zhang, Ruilun Chen, Delong Qiu, Dongguang Chen

**Affiliations:** 1 Department of Endocrinology, The Fifth Clinical College of Guangzhou University of Chinese Medicine, Guangdong Provincial Second Hospital of Traditional Chinese Medicine, Guangzhou, China; 2 Department of Neurology, Guangzhou Dongsheng Hospital (Guangzhou Geriatric Rehabilitation Hospital), Guangzhou, China; 3 Guangdong Provincial Engineering Technology Research Institute of Traditional Chinese Medicine, Guangzhou, China; 4 Guangdong Provincial Key Laboratory of Research and Development in Traditional Chinese Medicine, Guangzhou, China

**Keywords:** diabetic ketoacidosis, endogenous insulin secretion, insulin autoantibody, insulin therapy, subcutaneous insulin resistance, type 2 diabetes

## Abstract

**Background:**

Subcutaneous insulin resistance (SIR) is a rare condition characterized by impaired responsiveness to subcutaneous insulin despite preserved intravenous insulin efficacy. The coexistence of SIR with rapid loss of endogenous insulin secretion following exogenous insulin exposure has rarely been reported.

**Case presentation:**

A 47-year-old Chinese man initially classified as having type 2 diabetes (T2D) had preserved endogenous insulin secretion (random C-peptide, 4.59 ng/mL) and negative insulin autoantibody (IAA), glutamic acid decarboxylase antibody, and insulinoma-associated protein 2 antibody at diagnosis. Shortly after initiation of subcutaneous insulin therapy, he developed pruritic, erythematous, and indurated injection-site lesions with progressive hyperglycemia and subsequently presented with diabetic ketoacidosis. C-peptide became undetectable, whereas IAA increased to >175 IU/mL. Intravenous insulin consistently restored glycemic control, while several subcutaneous insulin preparations were initially ineffective, indicating a marked route-dependent difference in insulin responsiveness. Subcutaneous insulin responsiveness later recovered, first with biphasic protamine human insulin 40/60 and subsequently with insulin analogues, despite persistent IAA positivity. At recent follow-up, 14-day continuous glucose monitoring showed a mean glucose of 7.6 mmol/L, time in range of 80%, and time below range of 3%. A heterozygous *WFS1* variant (NM_006005.3:c.2147C>G, p.Ala716Gly) was identified as an exploratory finding of uncertain clinical significance.

**Conclusion:**

This case highlights the coexistence of rapid loss of endogenous insulin secretion and severe but reversible SIR following exogenous insulin exposure. Preserved intravenous insulin responsiveness and recovery of subcutaneous insulin efficacy despite persistent IAA positivity underscore the dynamic and route-specific nature of this phenotype. The underlying mechanism remains uncertain, and no causal relationship with the *WFS1* variant can be inferred. Recognition of this pattern may facilitate appropriate insulin delivery and longitudinal monitoring.

## Introduction

Insulin therapy remains a cornerstone of diabetes management; however, immune-related adverse reactions to exogenous insulin, although uncommon, represent clinically important complications beyond hypoglycemia. These reactions range from localized injection-site hypersensitivity to systemic antibody-associated responses, including exogenous insulin antibody syndrome (EIAS) (1). More recently, cases have been reported in which patients initially diagnosed with type 2 diabetes (T2D) developed a rapid loss of endogenous insulin secretion after initiation of exogenous insulin therapy (2).

Previous studies, primarily from Japanese cohorts, have reported that this clinical evolution often occurred in individuals carrying type 1 diabetes (T1D)-associated HLA class II genotypes (2, 3). Similar cases reported in Chinese populations have also been associated with classical T1D-related HLA backgrounds (4). However, the genetic background and biological mechanisms underlying this unusual clinical evolution remain incompletely understood.

Here, we report a patient initially classified as having T2D who developed rapid loss of endogenous insulin secretion and severe but reversible subcutaneous insulin resistance shortly after initiation of exogenous insulin therapy. Unlike several previously described cases, this patient did not carry the classical high-risk HLA class II genotypes reported in association with this phenotype. A heterozygous WFS1 variant was identified during genetic evaluation, although its clinical significance remains uncertain. This case highlights the unusual coexistence of rapid loss of endogenous insulin secretion and reversible route-specific insulin resistance after exogenous insulin exposure, while the clinical significance of the accompanying genetic findings remains uncertain.

## Case presentation

A 47-year-old Chinese man presented to the emergency department with a 1-day history of nausea, vomiting, and transient fever. During the 6 months preceding the diagnosis of diabetes, he had unintentionally lost approximately 8 kg. One month before admission, he developed progressive polydipsia and polyuria. He had no significant previous medical history and no family history of diabetes or other major hereditary disorders. He had three children, all of whom were reportedly healthy.

At the initial diagnosis, fasting plasma glucose was 14.11 mmol/L and glycated hemoglobin (HbA1c) was 11.7%. Endogenous insulin secretion was preserved, with a random C-peptide concentration of 4.59 ng/mL. Insulin autoantibody (IAA), glutamic acid decarboxylase antibody (GADA), and insulinoma-associated protein 2 antibody (IA-2A) were negative. Zinc transporter 8 antibody (ZnT8A) was not measured at this time. Based on the clinical presentation, preserved C-peptide secretion, and negative available islet autoantibodies, he was initially classified as having T2D. Reliable prediagnosis body weight and BMI data were unavailable; therefore, the degree of baseline insulin resistance could not be formally assessed.

Initial treatment consisted of subcutaneous biphasic insulin aspart 30/70 (24 units before breakfast and 22 units before dinner), combined with metformin (500 mg twice daily) and dapagliflozin (10 mg daily). Glycemic control initially improved after treatment initiation. However, approximately two weeks before admission, the patient developed pruritic, erythematous, and indurated lesions at multiple abdominal insulin injection sites. A skin biopsy was proposed during hospitalization to characterize the injection-site lesions; however, the patient declined the procedure, and histopathological evaluation was therefore unavailable. Despite adjustment and escalation of the subcutaneous insulin regimen, glycemic control deteriorated rapidly.

One day before admission, the patient developed nausea and vomiting. On admission, his BMI was 19.3 kg/m² and heart rate was 134 beats/min. Because he had lost approximately 8 kg during the preceding 6 months, the admission BMI may not reflect his premorbid body habitus. Multiple erythematous and indurated nodules were present at previous abdominal insulin injection sites (**Figure 1A**). A fruity odor was noted on physical examination. Laboratory evaluation confirmed severe diabetic ketoacidosis (DKA), with plasma glucose of 30.82 mmol/L, arterial pH of 6.86, and β-hydroxybutyrate of 13.63 mmol/L. At this time, serum insulin and C-peptide concentrations were below the assay detection limits, representing a striking decline from the random C-peptide concentration of 4.59 ng/mL documented before insulin exposure. IAA was markedly elevated (>175 IU/mL), whereas GADA and IA-2A remained negative.

**Figure 1:**
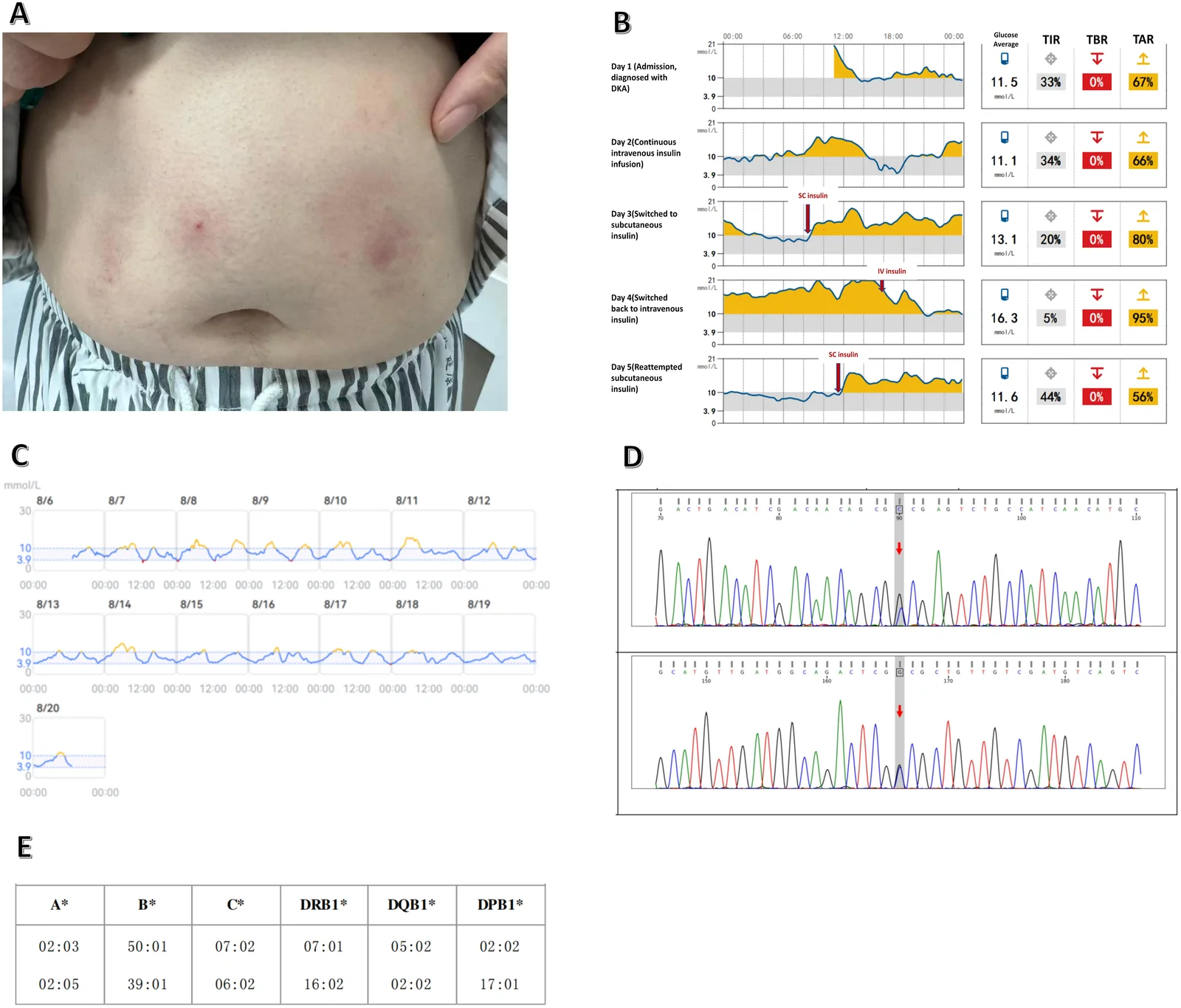
Clinical, glycemic, and genetic findings during the acute phase and long-term follow-up. **(A)** Pruritic, erythematous, and indurated lesions at abdominal insulin injection sites during the acute phase. Skin biopsy was not performed because the patient declined the procedure. **(B)** CGM profiles during the first 5 days of hospitalization showing preserved intravenous (IV) insulin responsiveness and recurrent hyperglycemia after transition to subcutaneous (SC) insulin. Yellow-shaded areas indicate glucose >10.0 mmol/L; arrows indicate changes in insulin administration route. **(C)** Fourteen-day CGM during long-term follow-up (August 6–20, 2026) while receiving SC insulin glargine and aspart, showing mean glucose 7.6 mmol/L, TIR 80%, TAR 17%, TBR 3%, and CV 34.2%. **(D)** Sanger sequencing confirming the heterozygous *WFS1* variant NM_006005.3:c.2147C>G (p.Ala716Gly); its clinical significance remains uncertain. **(E)** High-resolution HLA genotyping by next-generation sequencing. CGM, continuous glucose monitoring; IV, intravenous; SC, subcutaneous; TIR, time in range; TAR, time above range; TBR, time below range; CV, coefficient of variation; HLA, human leukocyte antigen.

DKA resolved after intravenous fluid replacement and continuous intravenous insulin infusion. When intravenous insulin was subsequently transitioned to subcutaneous insulin, including continuous subcutaneous insulin infusion with insulin aspart, marked hyperglycemia rapidly recurred despite repeated dose adjustments and changes in infusion sites. Reintroduction of intravenous insulin consistently improved glycemia (**Figure 1B**), demonstrating a marked route-dependent difference in insulin responsiveness.

During the acute phase, several subcutaneous insulin preparations, including human insulin and the insulin analogs glargine, degludec, lispro, and aspart, were sequentially attempted but initially failed to provide adequate glycemic control. Subsequently, a trial of subcutaneous biphasic protamine human insulin 40/60 combined with acarbose successfully maintained glucose concentrations at approximately 5–10 mmol/L. This response indicated that the impairment of subcutaneous insulin responsiveness was severe but not absolute and had already begun to recover. The marked impairment of subcutaneous insulin efficacy together with preserved intravenous insulin responsiveness supported a clinical diagnosis of severe subcutaneous insulin resistance (SIR).

Given the abrupt loss of endogenous insulin secretion and the unusual insulin-associated clinical course, immunological and genetic evaluations were performed. HLA typing identified DRB1*07:07 and DRB1*16:02, and DQB1*05:02 and DQB1*02:02 (**Figure 1E**). Whole-exome sequencing identified a heterozygous missense variant in WFS1 (NM_006005.3:c.2147C>G, p.Ala716Gly), which was subsequently confirmed by Sanger sequencing (**Figure 1D**). The variant was not reported in the gnomAD population database according to the clinical genetic analysis and was classified as a variant of uncertain significance. No functional evidence was available to establish a causal relationship with the observed phenotype.

During long-term follow-up, subcutaneous insulin responsiveness progressively recovered. IAA remained positive; IA-2A became transiently positive and subsequently reverted to negative, whereas GADA and ZnT8A were negative when assessed (**Table 1**). At the most recent assessment, the patient was treated with subcutaneous insulin glargine 15 U nightly and insulin aspart 15 U before each meal. Fourteen-day CGM monitoring from August 6 to August 20, 2026 (**Figure 1C**), with 100% sensor use, showed a mean glucose concentration of 7.6 mmol/L, time in range (3.9–10.0 mmol/L) of 80%, time above range of 17%, and time below range of 3%. The coefficient of variation was 34.2%, and the estimated HbA1c was 6.4%. Nine CGM-detected hypoglycemic episodes occurred, with a mean duration of 49 minutes. These findings demonstrated overall favorable time in range, although CGM-detected hypoglycemia remained present.

**Table 1:** Clinical course, immunological changes, and therapeutic evolution.

Phase	Timeline	Clinical course & key findings	Immunogenetics & lab response	Therapeutic intervention
Initial diabetes phenotype	1 mo prior (Oct 2024)	Polydipsia, polyuria; ~8-kg weight loss over 6 months. FPG 14.11 mmol/L; HbA1c 11.7%; C-peptide 4.59 ng/mL.	IAA (−), GADA (−), IA-2A (−); ZnT8A not measured at baseline.	SC biphasic insulin aspart 30/70 + metformin + dapagliflozin; initial glycemic improvement.
Injection-site reactions and deterioration	2 wk prior (Early Nov 2024)	Pruritic, erythematous, and indurated injection-site lesions; progressive hyperglycemia despite SC insulin adjustment.	Not reassessed at this stage.	Escalation of SC insulin failed to restore adequate glycemic control.
DKA and rapid loss of endogenous insulin secretion	Day 0 (Nov 19, 2024)	Severe DKA; glucose 30.82 mmol/L, pH 6.86, β-OHB 13.63 mmol/L; insulin and C-peptide below detection limits.	IAA >175 IU/mL; GADA (−), IA-2A (−).	IV fluids + continuous IV insulin; DKA resolved. Hyperglycemia recurred after transition to SC insulin.
Severe impairment of SC insulin responsiveness	In-hospital (Late Nov 2024)	Marked discrepancy between SC and IV insulin responsiveness.	HLA: DRB1*07:07 and DRB1*16:02; DQB1*05:02 and DQB1*02:02. *WFS1* NM_006005.3:c.2147C>G (p.Ala716Gly), heterozygous VUS	Several SC insulin preparations were initially ineffective; biphasic protamine human insulin 40/60 + acarbose subsequently achieved glucose ~5–10 mmol/L.
Recovery phase	1–3 mo (Jan-Feb 2025)	SC insulin responsiveness progressively improved; no recurrence of severe injection-site reactions.	C-peptide remained below detection limit; IAA (+); IA-2A transiently (+); GADA (−); ZnT8A (−) when assessed.	Transitioned to SC glargine + aspart, with gradual restoration of SC insulin efficacy.
Long-term follow-up	~21 mo (Aug 2026)	Current regimen: glargine 15 U nightly + aspart 15 U before meals. Fourteen-day CGM: mean glucose 7.6 mmol/L, TIR 80%, TBR 3%, CV 34.2%.	IAA remained positive; IA-2A reverted to negative; GADA and ZnT8A negative when assessed.	Sustained SC insulin responsiveness without recurrence of the acute phase of severe impairment in SC insulin responsiveness.

β-OHB, β-hydroxybutyrate; CGM, continuous glucose monitoring; CV, coefficient of variation; DKA, diabetic ketoacidosis; FPG, fasting plasma glucose; GADA, glutamic acid decarboxylase antibody; HLA, human leukocyte antigen; IA-2A, insulinoma-associated protein 2 antibody; IAA, insulin autoantibody; IV, intravenous; SC, subcutaneous; TBR, time below range; TIR, time in range; ZnT8A, zinc transporter 8 antibody.

ZnT8A was not measured before initiation of insulin therapy; therefore, pre-existing islet autoimmunity cannot be completely excluded.

## Diagnostic assessment

The diagnostic reasoning was based primarily on the temporal evolution of endogenous insulin secretion and the route-dependent difference in insulin responsiveness. The decline in random C-peptide from 4.59 ng/mL at the initial diagnosis to below the assay detection limit at the time of DKA indicated a rapid loss of endogenous insulin secretion. The subsequent marked elevation of IAA (>175 IU/mL), together with the injection-site inflammatory reactions, suggested an insulin-associated immune response; however, IAA detected after exogenous insulin exposure could not by itself establish autoimmune β-cell destruction. Recurrent hyperglycemia after transition from intravenous to subcutaneous insulin, together with reproducible improvement after reintroduction of intravenous insulin, supported severe but reversible subcutaneous insulin resistance.

## Discussion

In this report, we describe an unusual clinical phenotype characterized by rapid loss of endogenous insulin secretion and severe but reversible subcutaneous insulin resistance shortly after initiation of exogenous insulin therapy in a patient initially diagnosed with T2D. The key features included a decline in C-peptide from 4.59 ng/mL to below the assay detection limit, marked IAA elevation, injection-site reactions, and a reproducible discrepancy between subcutaneous and intravenous insulin responsiveness. Related phenotypes have been reported in insulin hypersensitivity followed by DKA and profound insulin deficiency, as described by Teo et al. (5), and in classic extreme subcutaneous insulin resistance with preserved intravenous insulin sensitivity, as reported by Soudan et al. (6). Unlike the prolonged and severe course in the latter report, subcutaneous insulin responsiveness in our patient progressively recovered. The main similarities and differences are summarized in **Table 2**. Thus, the distinctive feature of the present case is the coexistence of rapid loss of endogenous insulin secretion and severe yet reversible subcutaneous insulin resistance after insulin exposure.

**Table 2:** Comparison with previously reported related cases.

Feature	Teo et al., 2022 (5)	Soudan et al., 2003 (6)	Present case
Diabetes background	Longstanding T2D	Longstanding T1D	Initially classified as T2D
Insulin-related skin reaction	Localized hypersensitivity after insulin detemir	Not prominent	Pruritic, erythematous, indurated injection-site lesions
DKA/ketosis	Severe DKA	Recurrent DKA/persistent ketosis	Severe DKA
Endogenous insulin secretion	C-peptide undetectable	C-peptide undetectable	C-peptide 4.59 ng/mL initially → below detection limit
Insulin antibodies/islet autoimmunity	Insulin antibodies elevated; ICA transiently positive	No increase in circulating insulin antibodies reported	IAA >175 IU/mL after insulin exposure; IA-2A transiently positive
SC insulin responsiveness	Reduced and variable	Markedly impaired	Markedly impaired during the acute phase
IV insulin responsiveness	Preserved during DKA treatment	Preserved	Preserved
Route-specific insulin resistance	Not clearly established	Defining feature	Clear and reproducible
Course of SC insulin resistance	Persistent/variable	Severe and prolonged	Severe but reversible
Long-term treatment/outcome	MDI/CSII; persistent insulin dependence	IV and intraperitoneal insulin delivery required	SC insulin responsiveness recovered; SC insulin remained effective during follow-up
Key distinguishing feature	Insulin hypersensitivity followed by DKA and profound insulin deficiency	Classic extreme SIR with preserved IV insulin sensitivity	Rapid loss of endogenous insulin secretion with reversible SIR after insulin exposure

CSII, continuous subcutaneous insulin infusion; DKA, diabetic ketoacidosis; IA-2A, insulinoma-associated protein 2 antibody; IAA, insulin autoantibody; ICA, islet cell antibody; IV, intravenous; MDI, multiple daily injections; SC, subcutaneous; SIR, subcutaneous insulin resistance; T1D, type 1 diabetes; T2D, type 2 diabetes.

The present phenotype also differs from several established diabetes-related entities. EIAS is characterized mainly by insulin antibody-associated glycemic instability (1), whereas fulminant type 1 diabetes (7) is defined by rapidly progressive insulin deficiency and DKA but does not account for the route-specific difference in insulin responsiveness observed here. LADA (8) and SPIDDM (9) generally show a more gradual decline in β-cell function with persistent islet autoantibody positivity. Therefore, none of these entities alone fully explains the combined phenotype in this patient.

Previous reports of rapid loss of endogenous insulin secretion after insulin exposure have often involved HLA class II genotypes associated with T1D susceptibility (2–4). Our patient did not carry the classical high-risk HLA genotypes reported in those cases. Whole-exome sequencing identified a heterozygous *WFS1* missense variant (NM_006005.3:c.2147C>G, p.Ala716Gly), which was confirmed by Sanger sequencing. The variant was not reported in the gnomAD population database according to the clinical genetic analysis and was classified as a variant of uncertain significance. Given the absence of a personal or family history suggestive of a *WFS1*-related disorder and the lack of functional or segregation evidence, this finding should be regarded as exploratory rather than as evidence of a susceptibility mechanism. Accordingly, **Figure 2** summarizes the temporal clinical evolution following exogenous insulin exposure and does not imply a causal relationship between the WFS1 variant, IAA elevation, and the rapid loss of endogenous insulin secretion.

**Figure 2:**
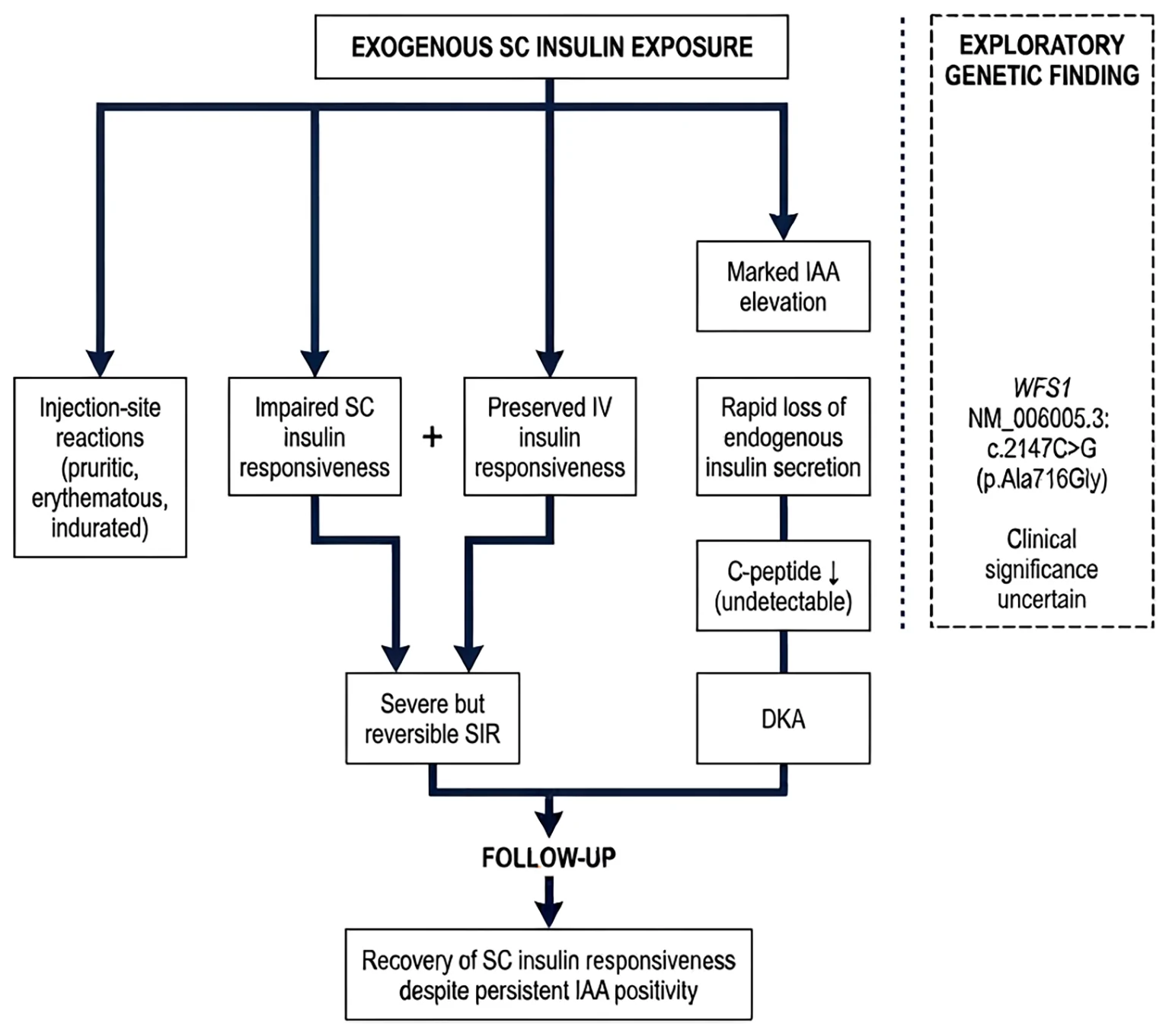
Clinical evolution following exogenous insulin exposure. The diagram summarizes the temporal association of injection-site reactions, marked IAA elevation, severe but reversible subcutaneous insulin resistance, and rapid loss of endogenous insulin secretion after initiation of subcutaneous insulin therapy. Subcutaneous insulin responsiveness subsequently recovered despite persistent IAA positivity. The *WFS1* p.Ala716Gly variant is shown as an exploratory genetic finding of uncertain clinical significance; no causal relationship is implied.

The mechanism underlying the transient route-specific impairment of insulin responsiveness in this patient remains uncertain. The reproducible improvement with intravenous insulin despite marked impairment of subcutaneous insulin efficacy supports a route-specific process rather than generalized insulin resistance. The marked elevation of IAA raises the possibility of an antibody-associated contribution to altered insulin kinetics or bioavailability (1, 5); however, the available data do not establish IAA as the primary mechanism of the subcutaneous insulin resistance. Notably, subcutaneous insulin responsiveness recovered despite persistent IAA positivity, arguing against a simple direct relationship between antibody positivity and the severity of route-specific insulin resistance. Previous reports have suggested heterogeneous mechanisms for subcutaneous insulin resistance, including altered local insulin degradation, sequestration, or absorption, but these abnormalities are not consistently demonstrable across cases (6, 10).

During long-term follow-up, recovery of subcutaneous insulin responsiveness permitted conventional subcutaneous insulin therapy without immunomodulation. Fourteen-day CGM showed a mean glucose concentration of 7.6 mmol/L, TIR of 80%, TBR of 3%, and a coefficient of variation of 34.2%, with nine CGM-detected hypoglycemic episodes. Thus, overall glycemic exposure was acceptable, although some glycemic variability and CGM-detected hypoglycemia remained. Because subcutaneous insulin efficacy had recovered and no persistent severe dysglycemia requiring immunomodulatory therapy was documented, immunomodulatory treatment was not initiated. Such treatment may be considered in selected refractory cases, but current evidence remains limited (5, 11).

This case has several limitations. First, ZnT8A was not measured before insulin initiation, and pre-existing islet autoimmunity therefore cannot be completely excluded. Second, histopathological confirmation of the injection-site reactions was unavailable because the patient declined skin biopsy. Third, no functional or segregation studies were performed to determine the clinical relevance of the *WFS1* p.Ala716Gly variant. In addition, insulin-antibody binding characteristics and local insulin degradation or absorption were not directly assessed. Concomitant dapagliflozin therapy represents a potential confounder for the occurrence of DKA (12), although it does not explain the abrupt loss of endogenous insulin secretion or the route-dependent difference in insulin responsiveness. Finally, as a single case report, the temporal association between exogenous insulin exposure, IAA elevation, rapid loss of endogenous insulin secretion, and subcutaneous insulin resistance cannot establish causality. Nevertheless, the reproducible discrepancy between subcutaneous and intravenous insulin responsiveness and the subsequent recovery of subcutaneous insulin efficacy provide clinically informative observations for this rare phenotype.

## Patient perspective

The patient reported that the most distressing period was the marked glycemic instability and need for intravenous insulin when subcutaneous insulin became ineffective despite escalating doses. At recent follow-up, he stated that he had accepted the need for long-term insulin therapy but remained interested in understanding the cause of this unusual phenomenon and whether additional treatment options might become available. He continues regular follow-up and remains actively involved in treatment decisions.

## Conclusions

This case highlights the coexistence of rapid loss of endogenous insulin secretion and severe but reversible subcutaneous insulin resistance following exogenous insulin exposure. Preserved intravenous insulin responsiveness and recovery of subcutaneous insulin efficacy despite persistent IAA positivity underscore the dynamic and route-specific nature of this phenotype. The underlying mechanism remains uncertain, and no causal relationship with the *WFS1* variant can be inferred. Early recognition of this pattern may facilitate appropriate insulin delivery and longitudinal monitoring.

## Data Availability

The original contributions presented in the study are included in the article. Further inquiries can be directed to the corresponding author.

## References

[1] Hu X Chen F . Exogenous insulin antibody syndrome (EIAS): a clinical syndrome associated with insulin antibodies induced by exogenous insulin in diabetic patients. Endocr Connect. (2018) 7:R47–R55. doi: 10.1530/EC-17-0309 29233817 PMC5776673

[2] Nakamura M Nishida W Yamada Y Chujo D Watanabe Y Imagawa A . Insulin administration may trigger pancreatic beta-cell destruction in patients with type 2 diabetes. Diabetes Res Clin Pract. (2008) 79:220–9. doi: 10.1016/j.diabres.2007.08.031 17950950

[3] Nishida W Nagata M Imagawa A Hanafusa T Ohashi J Takahashi K . Insulin administration may trigger type 1 diabetes in Japanese type 2 diabetes patients with type 1 diabetes high-risk HLA class II and the insulin gene VNTR genotype. J Clin Endocrinol Metab. (2014) 99:E1793–7. doi: 10.1210/jc.2014-1759 24971665

[4] Li XG Qi MY Li X Ping F . Exogenous insulin-associated autoimmunity and the emergence of double diabetes in type 2 diabetes. World J Diabetes. (2025) 16:109130. doi: 10.4239/wjd.v16.i9.109130 40980307 PMC12444274

[5] Teo CB Tan PY Lee SX Khoo J Tan JG Ang SF . Insulin allergy to Detemir followed by rapid onset of diabetic ketoacidosis: a case report and literature review. Front Endocrinol. (2022) 13:844040. doi: 10.3389/fendo.2022.844040 35350098 PMC8958006

[6] Soudan B Girardot C Fermon C Verlet E Pattou F Vantyghem MC . Extreme subcutaneous insulin resistance: a misunderstood syndrome. Diabetes Metab. (2003) 29:539–46. doi: 10.1016/s1262-3636(07)70069-1 14631332

[7] Hanafusa T Imagawa A . Fulminant type 1 diabetes: a novel clinical entity requiring special attention by all medical practitioners. Nat Clin Pract Endocrinol Metab. (2007) 3:36–45. doi: 10.1038/ncpendmet0351 17179928

[8] Buzzetti R Tuomi T Mauricio D Pietropaolo M Zhou Z Pozzilli P . Management of latent autoimmune diabetes in adults: a consensus statement from an international expert panel. Diabetes. (2020) 69:2037–47. doi: 10.2337/dbi20-0017 32847960 PMC7809717

[9] Shimada A Kawasaki E Abiru N Awata T Oikawa Y Osawa H . New diagnostic criteria (2023) for slowly progressive type 1 diabetes (SPIDDM): report from Committee on Type 1 Diabetes in Japan Diabetes Society (English version). Diabetol Int. (2024) 15:1–4. doi: 10.1007/s13340-023-00679-1 38264233 PMC10800325

[10] Schade DS Duckworth WC . In search of the subcutaneous-insulin-resistance syndrome. N Engl J Med. (1986) 315:147–53. doi: 10.1056/NEJM198607173150302 3523242

[11] Heinzerling L Raile K Rochlitz H Zuberbier T Worm M . Insulin allergy: clinical manifestations and management strategies. Allergy. (2008) 63:148–55. doi: 10.1111/j.1398-9995.2007.01567.x 18186805

[12] Umpierrez GE Davis GM ElSayed NA Fadini GP Galindo RJ Hirsch IB . Hyperglycemic crises in adults with diabetes: a consensus report. Diabetes Care. (2024) 47:1257–75. doi: 10.2337/dci24-0032 39052901 PMC11272983

